# Exercise Behaviour and Body Esteem of Gym-Goers in India

**DOI:** 10.5964/ejop.3687

**Published:** 2023-02-28

**Authors:** Ruhee Contractor, Deepa Rasquinha

**Affiliations:** 1 Department of Clinical Psychology, Manipal College of Health Professions, Manipal, India; University of Wroclaw, Wroclaw, Poland

**Keywords:** exercise dependence, body esteem, gym-goers, Indian study, sociocultural model

## Abstract

Exercise dependence is described as exercise which is harmful if engaged compulsively and excessively. The present study aims to investigate differences in categories of exercise behaviours and areas of body esteem in gym-goers in India across genders. The study used a cross-sectional design, and the sample consisted of 291 gym-goers (females = 146; males = 145) The Exercise Dependence Scale- 21 (EDS-21) and Body Esteem Scale-Revised (BES-R) was administered to the gym-goers in India after seeking informed consent. The obtained data were statistically analysed using descriptive statistics and multivariate analysis of variance. The results of the study indicated that there was a significant difference in the three categories of exercise behaviours and three areas of body esteem in male gym-goers. However, such similar differences were not found in female gym-goers. The differences found in body esteem for the male gym-goers in certain areas suggest how exercise has an impact on body image. For female gym-goers, we can see that irrespective of the category they belong to, there is no difference in the areas of body esteem. We can understand such findings with the sociocultural model of excessive exercise given by White and Halliwell (DOI: 10.1016/j.bodyim.2010.02.002) where perceived sociocultural pressure predicts excessive exercise, which is mediated by body image. Strengths and limitations of the study are discussed. Future research in India based on qualitative and longitudinal designs are warranted.

Exercise is seen as a healthy coping strategy and is associated with various benefits which can range from physical to psychological aspects of one’s life. There has been a plethora of studies that have proven the benefits (short and long-term) of regular physical exercise (Edwards, 2006; Penedo & Dahn, 2005; Salmon, 2001). However, another set of studies shows the adverse effects of exercise on our mental health and functioning. These studies usually look at the intensity of exercise and study exercise as a form of dependence in people’s lives (Allegre et al., 2006; Bamber et al., 2003; Cox & Orford, 2004).

## Exercise as a Negative Behaviour/ Exercise Dependence

According to Morgan (1968), excessive exercise can lead to several adverse outcomes. These outcomes can range from various physical injuries to problems in social and occupational functioning. An individual/individuals can also experience withdrawal symptoms during a break from exercise. Exercise behaviour is negative when a person starts experiencing significant dysfunction in at least two of the four primary areas of an individual’s life (Bamber et al., 2000) These areas are psychological, social and occupational, physical and behavioural.

Exercise dependence can lead to a series of potential pathological behaviours. These behaviours can lead individuals to experience events that alter their mood through which they achieve pleasure and then become dependent (Griffiths, 2005). Excessive exercise can lead to psychological burnout, along with disturbances in mood (Rejeski, 1994). Individuals with exercise dependence spend most of their time in exercise and related activities with increasing neglect in other areas of their lives. They may sometimes engage in multiple physical activities daily. There is also a decrease in other activities, and social interaction as exercise becomes a priority. Excessive exercise can lead to fatigue, injury, or illness. Even when there are multiple and repeated injuries individuals who have exercise dependence are unable to stop the exercise. They also engage in stereotypical and inflexible behaviour patterns while performing exercise and have rigid exercise schedules (Bamber et al., 2003).

According to Hausenblas and Downs (2002a) exercise dependence is when “physical activity is extreme in frequency and duration, relatively resistant to change, and is often associated with an irresistible impulse to continue exercising despite personal demands, fatigue, injury, or illness.” Exercise as an addiction can become a craving where an individual, for example, goes to the gym and exercises with high intensity for an extended period and neglects occupational, educational, and social areas of his/her life.

## Diagnostic Category for Exercise Dependence

Using the diagnostic criteria for substance abuse provided in DSM-IV, Hausenblas and Downs (2002b) have formulated the diagnosis for exercise dependence. Out of the seven dimensions, any three of them should meet the cut-off to diagnose a person as exercise dependent. Exercise dependence can be classified under behavioural addiction where an individual becomes obsessive and compulsive, and exercise causes dysfunction in a person’s life. A score of 15 and above obtained on the Exercise Dependence Scale-21 (EDS-21) is the cut-off for each of the dimensions (Hausenblas & Downs, 2002b). The seven dimensions are Tolerance, Withdrawal, Intention Effects, Lack of Control, Time, Reduction in Other Activity, and Continuance.

## Exercise and Body Esteem

Previous research suggests that regular physical activity leads to body image satisfaction and increased confidence in one's physical appearance (Cox & Orford, 2004). These people do not see exercise as enjoyment or a challenge but see it as a way to improve their looks and body. Several studies have shown that the rewards of looking attractive and appearing healthy have led people to alter their body shape. There is a rise in body image disturbance when such ideal forms cannot be achieved (Hausenblas & Fallon, 2002); Garner, 1997). There is a widespread notion that across genders, females experience greater body dissatisfaction (Lox et al, 2010). However, a similar trend of negative evaluation has also been seen in men (Watkins et al., 2008). It is interesting to note that the pressure to conform is not only felt by women, even males feel a similar strain, and there has been a tremendous rise in the male population ‘hitting’ the gym. There is a stark contrast in how women and men view body parts and shapes. For women, the ideal shape is an ‘ultra-thin, hourglass figure’ with a primary focus on hips, buttocks, thighs, or abdominal areas (Proshutina, 2012). The ideal male standard is increasing muscle mass and decreasing body fat to achieve a ‘toned and V-shaped physique’ where the focus is on the whole body looking broad and toned with muscles and abs. Males perceive their bodies as being smaller than what it is, and females perceive their bodies as being larger than what it is. The concern for men is both being too thin or fat, but for women, the primary concern is being fat (Lox et al., 2010).

Body esteem dissatisfaction, according to Johnson and Wardle (2005) can lead to various problems such as poor psychological adjustment, leads to excessive dieting, and symptoms of eating disorders. People with exercise dependence experience an increase in body satisfaction. The reasons for such a relationship could be due to the increase in one's attractiveness, muscle builds, weight loss/gain, feeling healthier and change in one’s physique, which can lead to such positive feelings. Understanding whether the areas of body esteem differ in different exercise behaviour groups especially that of individuals who are exercise dependent can be crucial in explaining body esteem as a contributing factor to excessive exercising. This information can further help prevent, modify, and remove these contributing factors from occurring.

## Sociocultural Model of Excessive Exercise

According to Seigel and Hetta (2001), there is an association between disturbances in body image and excessive exercise. The sociocultural model of excessive exercise given by White and Halliwell (2010) states that perceived sociocultural pressure has a direct relationship with a compulsive need for exercise, which is mediated by body image. In this model, perceived sociocultural pressure consists of pressure to lose weight, and build muscle, and the modelling of behaviour techniques to change the appearance. Body image consists of factors such as body dissatisfaction, body anxiety, and negative body affect. Thus, this model predicts pathological exercise behaviours rather than healthy ones. The model found specific gender differences. Boys had a greater need to engage in compulsive exercise than girls and reported engaging in higher frequency and duration of exercise than girls. This finding helps us understand “gendered choices of body shaping behaviours” (White & Halliwell, 2010, p. 232). It was also seen that body image disturbance in girls was higher than in boys. Body image disturbances were a more influential mediator of sociocultural pressure in girls than in boys.

Thus, the above model can be used to explain the study. The model will guide in understanding how body esteem can vary in different groups of exercise behaviours (at-risk for exercise dependence; nondependent-symptomatic, and nondependent-asymptomatic gym-goers). That is the difference between three categories of exercise and three areas of body esteem in male (sexual attractiveness, upper body strength and physical condition) and female (sexual attractiveness, weight concern and physical condition) gym-goers.

## Body Image Ideals: Indian Standards for Ideal Body for Men and Women

The current study focuses on the body esteem of young adults going to the gym in India. Tracing back to some of the studies that help us understand what the ideal standard of beauty is and how they are similar/different from that of the western culture.

The ideal beauty image for females typically includes a standard comparison with the “size-0 White” model. (Nagar & Virk, 2017). According to their study (2017), biological differences between Indian and Western ethnicities in terms of body shape, size and skin colour make these western standards of beauty highly challenging for Indian women. The comparison of Indian with Western standards of beauty is due to the advent of globalization and the popularisation of Western beauty standards by social media (Unnikrishnan & Prasad, 2016). According to researcher Rebecca Gelles (2011) the beauty standards of India are “narrowing and conforming to more international standards” and therefore these comparisons cause new physical and psychological challenges to the people, especially the youth in India. Rekha and Maran (2012) in their study on South Indian women found that people born during the globalization period are heavily influenced by the western dominated beauty standards.

A study conducted by Soohinda et al. (2020) found that males undergo sociocultural pressure and found that there is the internalization of both thinness and muscularity in Indian men. They found that exposure to media images of men displayed on magazine covers, movie posters, and social media propagating an idealized mesomorphic body has been shown to increase insecurity and body image concerns among men. Singh and Gadiraju (2020) also found that models that appear in popular media are approximately 20% lower than the normal body weight and there has been a significant increase in the portrayal of an athletic body for men in Indian advertisements.

Thus, the sociocultural model of excessive exercise can be understood within these parameters of body image dissatisfaction in India.

## Exercise Dependence and Body Image Studies

Understanding the prevalence of exercise dependence in India gives us an overall understanding of the behaviour in the sociocultural context. Previous studies have found a prevalence of 5.85% and it was found that exercise addiction was more prevalent in males than in females (Sharma et al., 2019).

A study aimed to understand whether exercise dependence would be predicted by physical self-concept (which is similar to body esteem in most aspects) among regular exercisers. The authors found significant correlations between exercise dependence symptoms and the domains of physical self-concept. Physical self-concept was also a strong predictor for exercise dependence in males and females (Oliva et al., 2013). Thus, this study helps us understand the relationship between gender, exercise dependence and body esteem.

A study conducted by Cook et al. (2013) examined the effect of gender as a moderating variable for exercise dependence. A gender difference was also found in exercise dependence symptoms where it was found that males scored higher on the total exercise dependence symptoms. It was also found that gender played a role in understanding the intensity of exercise and a gender difference was found in moderate and strenuous intensity exercise. The study, therefore, helps us understand gender differences in exercise dependence between genders.

A study was conducted by Ola and Singh (2016) on gym goers to find the relationship of gymming with mental health, body image satisfaction, aggression, and happiness where participants were compared on these variables. The results of the research found significant differences in the body satisfaction of gym goers as being more positive than that of controls. It was also found that gymming had a low positive correlation with body satisfaction. Thus, this research contributes to the existing literature which supports an increase in body esteem in gym goers.

A qualitative study conducted by Griffiths (2005) aimed to explore the positive and negative experiences of exercise. It was found that participants who were committed exercisers had functional reasons for their exercise behaviour as compared to at risk for exercise addiction group who gave negative reasons or causes for their negative exercise behaviour pattern.

The above literature gives a comprehensive look at all the variables related to the current study and the author has tried to build on this existing literature to understand exercise dependence and body esteem in gym goers across genders in the Indian context. There is also a gap in the literature regarding exercise dependence and gender differences in the body image of gym-goers in India. Thus, this study tries to reduce this existing gap in the literature to extend our sociocultural understanding of the relationships between exercise addiction and body esteem in gym goers.

## Objectives of the Study

As seen above, the prevailing emphasis on health and fitness in the youth has significantly led to the promotion of physical activity (Davis & Fox, 1993). There has been an increase in the number of people visiting gyms as it is one of the most convenient ways to promote physical exercise/activity. Therefore, the current study focuses on gym-goers.

The objective of the study is to investigate differences in body esteem among (a) at-risk for exercise dependence, (b) nondependent-symptomatic, and (c) nondependent-asymptomatic gym-goers. Body esteem is a multidimensional construct and is measured separately for both males and females. Therefore, the study contains two hypotheses:

1. H0: There will be no significant differences in body esteem among at-risk for exercise dependence, nondependent-symptomatic, and nondependent-asymptomatic male gym-goers.

2. H0: There will be no significant differences in body esteem among at-risk for exercise dependence, nondependent-symptomatic, and nondependent-asymptomatic female gym-goers.

## Method

### Participants

Male and female gym-goers in Karnataka, India who were between the ages of 18 to 40 years, did not have any physical disability, did not take part in any sort of fitness competitions and had joined the gym at least for the past three months were selected for the study. A total of 291 participants, 145 males and 146 females were the final sample. The mean age for males was 21 years (*SD* = 4.55) and the mean age for females was 22 years (*SD* = 3.60). The participants were divided into three groups of exercise behaviours: asymptomatic (Females = 57, Males = 45), symptomatic (Females = 63, Males = 75) and at-risk exercise dependent (Females = 26, Males = 30).

### Measures

#### Socio-Demographic Details

The socio-demographic datasheet constructed by the researcher assessed information regarding the participant’s age, occupation, education, gender, marital status, frequency of visit to fitness centres, use of substances, use of supplements, whether exercise prescribed by doctors due to a medical condition, number of hours spent exercising and time since they have joined the gym.

#### Exercise Dependence

The EDS-21 (Hausenblas & Downs, 2002b; Hausenblas & Fallon, 2002) was used to divide the participants into different categories of exercise behaviours. EDS-21 is a self-reported measure based on the DSM-IV criteria for substance dependence American Psychiatric Association (1994). A 6-point Likert-type scale was used to score EDS-21 where the scores ranged from 1 = Never to 6 = Always. The scores that fell in the dependent range were the higher scores of 5–6; scores of 3–4 were classified as symptomatic, and scores of 1–2 fell under the asymptomatic range. The scale differentiates between, a) individuals at-risk for exercise dependence, b) individuals that are nondependent-symptomatic, and c) individuals that are nondependent-asymptomatic. The internal consistency of the scale was high, demonstrated by α being 0.86. The range of coefficient alphas was from 0.78 to 0.92 (Hausenblas & Downs, 2002b).

#### Body Esteem

Body esteem was measured using the Body Esteem Scale-Revised (BES-R) (Frost et al., 2018). It is a self-report scale where participants report their feelings regarding 28 body parts. The scale indicates that body esteem is multidimensional and gender-specific. The dimensions are represented by the subscale scores, which are the summed-up responses for the items corresponding to each subscale. There are three subscales for women: sexual attractiveness, weight concern and physical condition, and three subscales for men: sexual attractiveness, upper body strength, and physical condition. It is a 5-point Likert scale where participants can rate their feelings ranging from 1 (strong negative feelings) to 5 (strong positive feelings about their different body parts. The internal consistency of the scale is high with values ranging from 0.82 to 0.94 for gender-specific subscales (Frost et al., 2018).

### Procedure

The researcher obtained permission letters from the respective gyms and collected data from the participants. To collect data, the researcher went to each gym for approximately ten days, preferably in the evenings for 3 hours. The researcher gave the participants information regarding the study and reassured them regarding the confidentiality of the data and study. The conditions were standardized for all participants and both questionnaires were administered at one time. The consenting participants were administered the EDS-21 (Hausenblas & Downs, 2002b) and the BES-R (Frost et al., 2018). After the administration of the scales, the participants were divided into three groups of nondependent asymptomatic, non-dependent symptomatic, and exercise dependent for further statistical analysis. The study was approved by the Institution Ethics Committee (Approval No. IEC-443-2019).

### Statistical Analysis

The resulting data were analysed using IBM Statistical Package for Social Sciences (SPSS version 16.0). A Multivariate Analysis of Variance (MANOVA) for males and females separately was conducted where the dependent variables were the three areas of body esteem, and the three categorical variables were exercise dependence, nondependent-symptomatic, and nondependent-asymptomatic.

## Results

### Sociodemographic Details

The socio-demographic details of the participants (see Table 1). The majority of the sample consisted of students (77.7%) with an undergraduate level of education (80.4%). Substance (12%) and supplement (26%) use was reported to be higher in the male participants. Medical reasons for exercise in male participants were primarily due to lower back pain and injury and female participants were prescribed to exercise due to Polycystic Ovary Syndrome (PCOS). On average, participants spent around six to eight hours (23.3%) at the gym per week.

**Table 1:** Socio-Demographic Characteristics of the Sample

Characteristic	Males (*N* = 145)	Females (*N* = 146)	All (*N* = 291)
Age			
*M*	21.35	22.46	21.91
*SD*	3.60	4.55	4.14
Education (%)			
Undergraduate	89.0	71.2	80.4
Postgraduate	8.9	27.4	18.2
Occupation (%)			
Student	79.5	75.3	77.7
Currently working	19.5	21.9	21.0
Marital Status (%)			
Married	93.8	8.9	7.2
Unmarried	6.2	91.1	92.8
Substance use (%)	12.3	8.2	10.3
Supplement use (%)	26.7	7.5	17.2
Prescribed exercise due to medical condition (%)	3.4	10.3	7.2
Number of hours spent in a week exercising			
0 to 2	5.5	10.3	7.9
2 to 4	4.8	12.3	8.6
4 to 6	19.2	23.3	21.3
6 to 8	22.6	24	23.3
8 to 10	13.7	11.6	12.7
10 to 12	17.8	9.6	13.7
12 to 14	9.6	6.2	7.9
14 to 16	3.4	1.4	2.4
Above 16	2.7	1.4	2.4
Number of months since the participants joined the gym		
3 to 11	55.9	77.4	66.6
12 to 48	37.2	18.5	27.8
49 to 120	6.9	4.1	5.5

### Exercise Behaviours and Body Esteem in Female Gym-Goers

A MANOVA was conducted to investigate differences in three areas of body esteem (sexual attractiveness, weight concern, and physical condition) and categories of exercise behaviours (non-dependent asymptomatic, non-dependent symptomatic, and at-risk exercise dependent) for female gym-goers. Table 2 shows the means and standard deviations of each area of body esteem with the three categories.

**Table 2:** Mean Scores, Standard Deviations and Sample Size for Female Gym-Goers Between Categories of Exercise Dependence and Areas of Body Esteem

Area of BES-R	Category of EDS-21	*M* (*SD*)	*N*
Sexual Attractiveness			
	Asymptomatic	33.28 (5.3)	57
	At-risk exercise dependent	34.11 (5)	26
	Symptomatic	31.91 (5.8)	63
	Total	32.84 (5.5)	146
Weight Concern			
	Asymptomatic	26.97 (7)	57
	At-risk exercise dependent	25.96 (8.1)	26
	Symptomatic	25.32 (7.8)	63
	Total	26.08 (7.5)	146
Physical Condition			
	Asymptomatic	22.46 (4.6)	57
	At-risk exercise dependent	22.54 (6)	26
	Symptomatic	21.06 (4.9)	63
	Total	21.87 (5)	146

The Box’s Test of Equality of Covariance Matrices test was conducted, and, in this study, the data set met the assumption (Box’s *M* = 7.539, *p* = .840). Bartlett’s Test of Sphericity was conducted, and a statistically significant outcome was obtained (approximate χ2 = 165.56, *p* < .001). After meeting the following assumptions, a MANOVA was calculated (see Table 3) and was found to be not statistically significant (*p* = .396) based on Pillai’s Trace test. A Pillai’s trace test was chosen as it is one of the best options to deal with an uneven sample size (Diaz-Garcia & Caro-Lopera, 2008). Thus, the hypothesis of the study that there will be no significant differences in body esteem among at-risk for exercise dependence, nondependent-symptomatic, and nondependent-asymptomatic female gym-goers was retained.

**Table 3:** Multivariate Analysis of Variance (MANOVA) Between Categories of Exercise Dependence and Areas of Body Esteem for Female Gym-Goers

Variable	Value	*F*	*p*	Observed power
Categories Pillai’s Trace	0.43	1.046	.396	.412

### Exercise Behaviours and Body Esteem in Male Gym-Goers

A MANOVA was conducted to find out the difference in three areas of body esteem (sexual attractiveness, upper body strength, and physical condition) and categories of exercise behaviours (non-dependent asymptomatic, non-dependent symptomatic, and at-risk exercise dependent) for male gym-goers. Table 4 shows the means and standard deviations of each area of body esteem with the three categories.

**Table 4:** Mean, Standard Deviations and Sample Size for Male Gym-Goers Between Categories of Exercise Dependence and Areas of Body Esteem

Area of BES-R	Category of EDS-21	*M* (*SD*)	*N*
Sexual Attractiveness			
	Asymptomatic	29.400 (4.9)	45
	Symptomatic	30.171 (4.9)	70
	At-risk exercise dependent	31.67 (6.2)	30
	Total	30.24 (5.3)	145
Upper Body Strength			
	Asymptomatic	18 (4.5)	45
	Symptomatic	20.03 (3.6)	70
	At-risk exercise dependent	19.73 (3.5)	30
	Total	19.34 (4)	145
Physical Condition			
	Asymptomatic	35.62 (8.2)	45
	Symptomatic	39.5 (6)	70
	At-risk Exercise dependent	38.4 (8.6)	30
	Total	38.07 (7.5)	145

The Box’s Test of Equality of Covariance Matrices test was conducted, and, in this study, the data set met the assumption (Box’s *M* = 7.539, *p* = .840). Another test, Bartlett’s Test of Sphericity, was conducted. A statistically significant outcome was obtained (approximate χ2 = 171.852, *p* < .001). After meeting the following assumptions, the MANOVA was calculated and was found to be significant (see Table 5). The hypothesis stated that there would be no significant differences in body esteem among exercise dependent, non-dependent-symptomatic, and non-dependent-asymptomatic male gym-goers, was rejected.

**Table 5:** Multivariate Analysis of Variance (MANOVA) Between Categories of Exercise Dependence and Areas of Body Esteem for Male Gym-Goers

Variable	Value	*F*	*p*	Observed power
Categories Pillai’s Trace	0.90	2.221	.041	0.779

There were three areas of body esteem, measured from the BES-R Scale (sexual attractiveness, upper body strength, and physical condition). Table 6 shows the univariate test results. The test showed significant differences across categories (non-dependent asymptomatic, non-dependent symptomatic and exercise dependent) on Upper Body Strength (UBS) (*F* = 3.83, *p* < .05) and Physical Condition (PC) (*F* = 3.88, *p* < .05).

**Table 6:** One-Way ANOVA for Male Gym-Goers Between Categories of EDS-21 and Areas of BES-R

Source	Dependent variable	*SS*	*df*	*MS*	*F*	*p*	Observed power
Categories of EDS-21	SA	93.14	2	46.57	1.70	.19	0.35
	UBS	118.63	2	59.32	3.83	.02	0.69
	PC	416.03	2	208.01	3.88	.02	0.69
Error	SA	3885.41	142				
	UBS	2191.81	142				
	PC	7613.28	142				
Total	SA	136587.0	145				
	UBS	56534.0	145				
	PC	218170.0	145				

*Note*. SA = Sexual Attractiveness, UBS = Upper Body Strength, PC = Physical Condition.

Table 7 shows Duncan’s homogenous subtests. The post hoc test results suggested that upper body strength differed significantly between Non-dependent asymptomatic (*M* = 18.00, *SD* = 4.54) and Exercise dependent (*M* = 19.73, *SD* = 3.48) groups and between Non-dependent asymptomatic (*M* = 18.00, *SD* = 4.54) and Non-dependent symptomatic groups (*M* = 20.29, *SD* = 3.6). There was no significant difference seen between Exercise dependent (*M* = 19.73, *SD* = 3.48) and Non-dependent symptomatic groups (*M* = 20.29, *SD* = 3.6). The post hoc test results suggested that physical condition in males differed significantly between Non-dependent symptomatic (*M* = 39.50, *SD* = 6) and Non-dependent asymptomatic (*M* = 35.62, *SD* = 8.24). There was no significant difference seen between Exercise dependent (*M* = 38.40, *SD* = 8.56) groups, and between Non-dependent asymptomatic (*M* = 35.62, *SD* = 8.24), and Exercise dependent (*M* = 38.40, *SD* = 8.56) groups and Non-dependent symptomatic groups (*M* = 39.50, behaviours *SD* = 6).

**Table 7:** Post-Hoc Duncan’s Homogenous Subtests for Upper Body Strength and Physical Condition for Male Gym-Goers

		Upper Body Strength Subset		Physical Condition Subset	
Category of exercise behaviour	N	1	2	1	2
Asymptomatic	45	18.00		35.62	
Exercise dependent	30		19.73	38.40	38.40
Symptomatic	70		20.03		39.50
*p*		1.00	.73	.081	.49

## Discussion

The researchers used a cross-sectional design in the study. The study aimed at investigating the differences between the three areas of body esteem and three categories of exercise behaviours in male and female gym-goers in India. The categories were formed by dividing the sample into three groups based on the scores obtained in the EDS-21 Scale.

The socio-demographic findings of the study are summarized. Substance use was higher in males than in females. However, there could be an under-reporting of substance use due to social desirability. The participants were selected on convenience and availability, influencing the demographic characteristics of the study. Therefore, the mean age of the participants was found to be 22 years across genders. Past studies also reveal that exercise dependence may be more prevalent in younger, particularly college-age students (Garman et al., 2004).

### Exercise Behaviours and Body Esteem of Female Gym-Goers

It was found that the differences between the areas of body esteem among different exercise behaviours of women were not different. Therefore, we accept the hypothesis stating “There will be no significant differences in body esteem among at-risk for exercise dependence, non-dependent-symptomatic, and non-dependent-asymptomatic female gym-goers”.

The findings can be compared to the past studies conducted on exercise dependence in females. As seen in the studies by (Nagar & Virk, 2017; Rekha & Maran, 2012; Unnikrishnan & Prasad, 2016) Indian women post globalization are now moving towards comparing their bodies with that of Western beauty ideals for women. Therefore, the present study will be comparing it to studies in the western culture studying exercise behaviours and body image in females. In a study conducted by Hausenblas and Downs (2002c), they found that primary exercise dependence symptoms marginally predicted body satisfaction in university students (Mean age = 20.26). Loland (2000) found that exercise behaviour did not predict body satisfaction in young females. As the mean age for the population was 22 years, the findings can be compared. In a study conducted on nutrition students, the authors suggested that students with a normal BMI would choose a lower ideal body weight and desire to weigh less due to social pressure or the desire to keep a fit image (Arroyo et al., 2010). Other studies have also shown that the stigma, discrimination, disapproval, and societal pressure faced by overweight girls and women creates a pressure to comply with the demands of fitting in the ideal body image (Weinberger et al., 2016). Thus, irrespective of the category of exercise behaviour they fall into, the body esteem is not significantly different for any of the groups. These findings could also be explained from the sociocultural point of view by looking at body image ideals for women. As stated in the model (White & Halliwell, 2010) body image disturbances were a more influential mediator of sociocultural pressure in girls than in boys but that boys had more compulsive need to exercise than girls, and they reported engaging in more exercise than girls. This finding helps us understand gendered choices of body shaping behaviours. Therefore, we see that sociocultural conditions play a central role in the development of body esteem.

### Exercise Behaviours and Body Esteem of Male Gym-Goers

The hypothesis for male gym-goers which states, “There will be no significant differences in body esteem among at-risk for exercise dependence, nondependent-symptomatic, and nondependent-asymptomatic male gym-goers” was rejected (see Table 5). Hausenblas and Downs (2002c) found similar results where male participants reported greater body satisfaction when they had reported harmful and excessive exercise behaviours. Exercise behaviour was the strongest predictor for body satisfaction in their study. Past research conducted by Watkins et al., (2008) has also found strong associations of body image and physical activity as exercise improves physical appearance and, therefore, a person’s body image. The “Affect regulation hypothesis” of Exercise dependence given by Hamer and Karageorghis (2007) can explain the above findings. The hypothesis states that exercise acts as a “positive effect enhancer,” which can help a person to develop and sustain positive feelings towards oneself. There is an increase in body satisfaction among people with exercise addiction. This increase could be due to the increase in one’s attractiveness; muscle builds, weight loss/gain, feeling healthier, greater stamina, change in one's physique, which can lead to such positive feelings (Cusumano & Thompson, 1997). Thus, male gym-goers may use exercise to improve their body image. Muscle building is now the societal ideal for a perfect male body, and it also becomes a factor to where they can show their strength and masculinity (Proshutina, 2012). This desire to fit into the societal ideal would place individuals at a higher risk for exercise dependence. These findings corroborate with the sociocultural model given above (White & Halliwell, 2010) and the societal ideal beauty standards for men in India (Singh & Gadiraju, 2020; Soohinda et al., 2020).

Comparing the results of the post hoc (see Table 7) it was found that sexual attractiveness was not significantly different among the three groups (at-risk for exercise dependence, nondependent-symptomatic, and nondependent-asymptomatic male gym-goers). This finding could be because most of the items on BES-R for sexual attractiveness are not majorly altered due to exercise (Frost et al., 2018). Thus, the role of sexual attractiveness is seen as independent when linked to exercise dependence. Meanwhile, the other two areas (upper body strength, and physical condition) were linked to exercise dependence.

Upper body strength was significantly higher in non-dependent symptomatic gym-goers and at-risk exercise dependent gym-goers (see Table 7). They were significantly different from nondependent asymptomatic gym-goers, whose mean was lower than theirs. The results of higher body satisfaction could be because the ideal body for males is lean and muscular which is partly determined by upper body strength and is difficult to achieve with just regular exercise (Hausenblas & Downs, 2002c). Upper body strength is improved by making the muscles bigger and broader. Muscle building is now the societal ideal for a perfect male body, and it also becomes a factor to show one's strength and masculinity. Thus, the means were higher for the at-risk exercise dependent and non-dependent symptomatic male gym-goers as a way of improving one's body esteem.

The mean of the scores on the Physical Condition subscale was significantly higher in the nondependent symptomatic group while comparing it with the other two groups who had no significant differences between them (see Table 7). This result could be explained by looking at the qualitative aspects of excessive exercise where Warner and Griffiths (2006) found that participants who reported health and fitness as a positive experience of exercise did not meet the criteria for exercise dependence (which is a score below 24 on the Exercise Addiction Inventory). Excessive exercise can have adverse effects such as fatigue, injury, pain, which could also affect one's physical condition. Thus, the perception of one's physical condition could be affected due to excessive/harmful exercise.

Thus, it can be seen that the areas of body esteem such as upper body strength and physical condition which can be mediated by exercise have shown a significant difference in the three exercise behaviour groups of male gym-goers.

### Strengths of the Study

The study adds to the existing literature on exercise behaviours, exercise dependence and body esteem in India. Few studies have looked at exercise dependence, and body esteem in gym-goers and this study gives insight into how the general population and not athletes or bodybuilders differ in body esteem and exercise dependence (Ganesan et al., 2018; Rajagopalan & Shejwal, 2014; Sharma et al., 2019; Soohinda et al., 2020) Exploration of the exercise behaviours in gym-goers in India with a sociocultural understanding of the findings is a notable contribution of the study. The study has also assessed different areas of body esteem which helps widen the scope of understanding of how exercise is related to the different areas. Understanding body esteem with exercise dependence is crucial in discerning the underlying causes of exercise behaviours, and this information can help prevent, modify, and remove these contributing factors from occurring. The differences found in body esteem for male gym-goers in certain areas suggest how exercise has an impact on body image and how there could be a rise in exercise behaviour due to decrease in body dissatisfaction. For prevention as well as treatment, it is essential to understand who will be at risk of developing harmful exercise behaviours because of alteration of their body esteem. The findings of the study will help in understanding the same.

### Limitations of the Study

Even though the findings of the study provide a better understanding of differences in body esteem in different categories of exercise behaviour, it is essential to mention the notable limitations of the study. First, the time when the data was collected was in the evening for three hours on weekdays at various gyms according to permissions granted for access from the gyms. Due to this, there was a loss of a significant number of participants in the morning and afternoons, as regular exercisers are likely to start exercising early in the mornings, which could be a possible limitation. Second, the number of participants in all three categories of exercise behaviour was not the same, and this could have affected the statistical analysis. Third, the demographics of the sample indicated that the group consisted of primarily young university students, which makes the generalizability of the results limited. One more limitation was the design of the study. It was a cross sectional design which does not help us understand the cause-and-effect relationship of variables.

### Conclusion

The findings of the study show that there is a significant difference in the three categories and three areas of body esteem in the male gym-goers. The current research showed that at-risk exercise dependent male gym-goers had a better perception of their upper body strength compared to nondependent asymptomatic male gym-goers. Nondependent symptomatic male gym-goers had a better impression of their upper body strength and physical condition compared to nondependent asymptomatic male gym-goers. Future research could use longitudinal, qualitative designs to objectively assess body esteem and exercise behaviours. Further studies could also look at the role of such a diverse culture like India in shaping ideas of body esteem and exercise behaviours.
